# Context-Specific Resilience Through a Cultural Lens: Social-Ecological Factors Among Chinese Families of Children with Neurodevelopmental Disabilities

**DOI:** 10.1007/s10803-024-06605-x

**Published:** 2024-10-22

**Authors:** Xiaolu Dai, Yves Cho Ho Cheung, Xiaoyu Zhuang, Elvis Fong Wing Ng, Daniel Fu Keung Wong

**Affiliations:** 1 Department of Social Work, Hong Kong Baptist University, 15 Baptist University Road, Baptist University Road Campus, Kowloon Tong, Hong Kong; 2 Heep Hong Society, 133 Hoi Bun Road, Kwun Tong, Hong Kong

**Keywords:** Social ecological resilience, Neurodevelopmental disorders, Parenting stress, Hong Kong families

## Abstract

With the rise in attention towards Neurodevelopmental Disorders in Hong Kong and worldwide, understanding the role of social ecological resilience in reducing parenting stress and enhancing child outcomes is crucial, particularly within the unique cultural context of Chinese families. This study utilized a social ecological model to examine resilience factors at individual and interpersonal levels among Hong Kong parents of children with Neurodevelopmental Disorders. It also compared the stress and resilience conditions and differential functions of social ecological resilience between parents with and without children diagnosed with Neurodevelopmental Disorders. A sample of 447 parents of children with and without Neurodevelopmental Disorders were assessed by a newly developed Social Ecological Resilience Scale, along with measures of parenting stress and the internalizing and externalizing behaviors of their children. Independent sample t-tests showed that Chinese parents of children with Neurodevelopmental Disorders report significantly higher parenting stress and more internalizing and externalizing difficulties in their children compared to those without Neurodevelopmental Disorders. Multiple linear regression analyses indicated that enhanced social ecological resilience among parents predicted improved parenting stress and better outcomes in children’s behaviors. Notably, social ecological resilience factors showed varying effects between parents with and without children diagnosed with Neurodevelopmental Disorders. These findings highlight that both individual and interpersonal resilience factors among parents predicted improved parenting stress and better outcomes in children’s behaviors and certain resilience factors may be contextually motivated. Policy makers and practitioners should consider developing context-specific strategies and programmes to help the focal target group.

## Introduction

Neurodevelopmental disorders (NDDs) constitute a spectrum of conditions characterized by developmental impairments in the brain and/or central nervous system, such as intellectual disability (ID), autism spectrum disorder (ASD), and attention deficit hyperactivity disorder (ADHD). Emerging typically in early life and often enduring throughout one’s lifetime, NDDs can significantly impact various aspects of daily life, including learning, behaviour, and communication (Thapar et al., 2017). In Hong Kong, the number of children with NDDs has increased in recent years. Around 90% of NDDs are identified as ADHD, ASD, or learning difficulties, with diagnoses rising from the preschool years and peaking in the early primary school years (around ages 5 to 8; Child Assessment Service, 2020; 2023).

Children with neurodevelopmental disorders (NDDs) often exhibit internalising and externalising behaviours that are challenging to manage (Danielsson et al., 2024). Studies have consistently indicated that these children display higher levels of internalising (e.g., anxiety and depression) and externalising behaviours (e.g., aggression, hyperactivity, and defiance) compared to their typically developing peers (Bauminger et al., 2010; Mayes et al., 2012). These behaviours not only result in profound negative impacts on the children’s psychosocial and cognitive functions (Arim et al., 2015; Hill et al., 2014) but also lead to diminished quality of life (Armstrong et al., 2015). Furthermore, managing the children’s behavioural difficulties on a daily basis becomes a persistent source of stress for parents, intensifying the challenges faced by families of children with NDDs (Bauminger et al., 2010).

Parental stress is defined as a psychological response that arises when parents face demands that do not align with their own expectations or those of society, or when they lack the necessary resources to meet these demands (Holly et al., 2019). Child characteristics have been identified as one of the key factors influencing the intensity of stress experienced by parents (Abidin, 1992). Examples include managing the child’s emotional and behavioural problems; navigating educational systems that may lack provisions for inclusive education; dealing with societal misunderstandings or stigma; and enduring significant financial pressures (Tait et al., 2016). It is not surprising that parents of children with NDDs report higher levels of parenting stress compared to parents of typically developing children (Bonis, 2016; Hassall et al., 2005). The consequences of high parenting stress are manifold, leading to disruptions in parental psychological functioning (Choi et al., 2023) and poor parenting practices (Mak & Kwok, 2010). Heightened parenting stress, in turn, was correlated with more emotional and behavioural difficulties in children (Dao et al., 2021).

Given the substantial stressors faced by families of children with NDDs, it is essential to identify protective factors that may buffer these adverse impacts. This study explores the role of resilience as a key protective factor in helping parents manage the stress associated with raising a child with NDDs. By focusing on the social ecological model of resilience, we aim to provide a more comprehensive understanding of how resilience operates at multiple levels—individual, familial, and community—and how these levels interact to influence both parent and child outcomes.

### Resilience as a Protective Factor

Resilience is recognized as an important protective factor that helps parents cope with the stress related to raising a child with NDDs (Peer & Hillman, 2014). It is defined as the ability of an individual to bounce back and maintain competence despite significant risk exposure (Smith, 2006). Researchers have found that parents with NDD children who demonstrate resilience are more likely to acknowledge their children’s limitations and are actively seeking effective ways to support them. In turn, this alleviates their children’s behavioral symptoms (Deater-Deckard, 2004; Lee et al., 2015; Cantero-García & Alonso-Tapia, 2018). In addition, parental resilience also reduces the stress levels experienced by parents with children with NDDs (Montirosso et al., 2021).

However, the concept of resilience has been a subject of debate in recent years. Researchers critiqued the heavy focus on individual psychological characteristics for neglecting the broader eco-systemic influences on resilience (Meisels & Shonkoff, 2000; Waller, 2001). Some researchers argued that environmental factors and accessible resources play a crucial role in sustaining resilience amid adversity (Ungar et al., 2013). Responding to these critiques, some researchers have proposed a social ecological model of resilience (SER) that underscores the critical impact of social and environmental contexts on resilience (Bronfenbrenner & Ceci, 1994; Ungar et al., 2013). Focusing on the ability to access resources that support well-being and the capacity to negotiate for these resources in culturally meaningful ways (Ungar, 2011), the SER framework is pivotal for understanding how various personal, social, environmental, and cultural factors interplay in resilience, particularly for crafting interventions aimed at at-risk groups.

### The Social Ecological Model of Resilience in a Chinese Familial Context

Drawing on the seminal work of Bronfenbrenner and Ceci (1994), our research team has adopted the concept and identified six factors nested within a three-level social ecological model of resilience for Chinese families (Wong et al., 2023). Comprised of *individual*, *family and microsystem*, as well as *interpersonal and ecosystem* levels of resilience, this framework offers a structured approach to understand resilience factors that potentially contribute to enhancing the health and mental health of parents and their children with NDDs within Chinese cultural context.

#### Individual Level

In our previous work, *emotional flexibility*,* coping ability*, *self-kindness*, and *common humanity* were identified as resilience factors at an individual level. *Emotional flexibility* refers to the ability to adapt their emotional responses according to the specific demands of different situations (Aldao et al., 2015). Literature has evidenced the important roles of emotion regulation, flexibility, and coping abilities in resilience (Martin et al., 2015). Additionally, *self-kindness* and *common humanity* can be regarded as an adaptive process that enhances psychological resilience and mental wellbeing (Neff, 2003). While ‘self-kindness’ enables parents to evaluate their situations without self-criticism (Reid et al., 2014), ‘common humanity’ helps parents recognize that failure and suffering are universally experienced (Wong et al., 2016).

In summary, individual factors such as emotional flexibility, coping ability, self-kindness, and common humanity form the foundation of resilience. However, these personal attributes do not exist in isolation; they are significantly influenced by the immediate family environment, where the support and dynamics of family members play a crucial role.

#### Family and Microsystem Levels

Family support is essential for the development and resilience of family members. Mutual support among family members boosts confidence in coping and fosters healthy family functioning (Walsh, 2003). A previous study showed that strong supportive families can mitigate the negative emotional impacts of life’s adversities (Bae & Kung, 2000). Interestingly, family support is said to be of greater importance for Chinese families, where there is a collectivist culture emphasizing family harmony and pride. Chinese families often prefer the support of familial networks over seeking external help (Bond, 2010). Thus, strong family support might be particularly important in building resilience and buffering stress in families of children with NDDs in Chinese society.

Notably, however, families do not operate in isolation; they are embedded within a larger social ecosystem. This leads us to the interpersonal and exosystem levels, where external support systems such as friends, neighbors, and community services add an additional layer of protection for families facing adversity.

#### Interpersonal and Exosystem Levels

Social support refers to the assistance received from broader social networks such as friends, groups, and communities (Lin et al., 1979). Social support serves as a foundational support system for individuals facing life’s challenges (Canavan & Dolan, 2000) and moderating stress against various vulnerabilities (Ozbay et al., 2007). For example, access to community resources (e.g., neighborhood network and public service), has been identified as a key exosystem factor in strengthening resilience and well-being, leading to positive outcomes for disadvantaged families (Aronowitz & Morrison-Beedy, 2004; Ungar et al., 2013). In Chinese culture, the importance of neighborhood support is reflected in the saying, “A good neighbor outmatches a distant relative”, highlighting the significance of mutual and community assistance in fostering family resilience. Hence, there is value in exploring whether social support as a social ecological resilience factor would help parents feel less stressed when managing their children with NDDs.

In sum, the social ecological model of resilience provides a framework for recognizing various factors within an individual’s social ecological system at different levels that can affect their health and well-being. However, since the social ecological model of resilience is a rather novel concept, there is a lack of research using this framework to explore the social and ecological resilience factors influencing parenting stress and children’s internalizing and externalising behaviours among parents of children with NDDs, especially in Chinese populations. Such exploration would facilitate the identification of not just individual and personal protective factors, but salient interpersonal factors that may affect parents’ and children’s health and wellbeing.

### The Current Study

This study used a social ecological resilience perspective in examining different levels of factors in an individual, his/her family and microsystem, as well as interpersonal and exosystem levels that could potentially influence the mental health of parents and their children with NDDs in Hong Kong. In particular, it examined the impacts of different social ecological factors of resilience on parenting stress and children’s internalising and externalising difficulties. It also compared the stress and resilience conditions and differential functions of social ecological resilience between parents with and without children diagnosed with NDDs.

It was hoped that such exploration would provide insight and recommendations into developing programmes that could build protective resilience factors for parents of children with NDDs in Hong Kong and elsewhere. We had the following hypotheses:

#### Hypothesis 1

Parents with higher social ecological resilience would have positive impacts on the parenting stress and children’s internalising and externalising behaviours;

#### Hypothesis 2

The parents of children with NDDs would have higher parenting stress and lower social ecological resilience than those parents of children without NDDs; and.

#### Hypothesis 3

Different social ecological resilience would have differential impacts on the parenting stress and children’s internalising and externalising behaviours between parents with and without children diagnosed with NDDs.

### Methods

#### Participants

An a priori power analysis using G*Power version 3.1.9.7 found a sample size of 188 would be needed to achieve α = 0.05 and power = 0.90 (Faul et al., 2007) under the effect size calculated from the between-factor correlations listed in Wong et al. (2023). To meet our research objectives, we aimed to recruit 188 parents of children diagnosed with NDDs and 188 parents of children without NDDs. The inclusion criteria are: (1) parents aged 18 years or above, (2) could understand Chinese language and fill out a questionnaire without assistance, and (3) had at least one child aged younger than 12 years. Parents who had two or more children within the age range were asked to report the child showing more behavioral difficulties.

### Procedures

Participants were recruited from one of the largest nonprofit organizations in Hong Kong that serves children and young people with diverse needs, mainly NDDs. This organization provides assessment, guidance, training, and family support services through its extensive network of training centres, which includes 17 Special Child Care Centres, 9 Mix-Mode Centres, and 7 Parents Resource Centres, etc. Social workers at these centres invited parents who met the inclusion criteria to participate in the survey.

In the survey, parents were asked to report if their children had any of the following official NDDs diagnoses according to the DSM-5-TR (American Psychiatric Association, 2022): communication disorders, ASD, ADHD, specific learning disorder, intellectual developmental disorders, global developmental delay (if children were under 5 years of age), motor disorders, and tic disorders. A total of 447 valid questionnaires were collected between February and March 2022. Of these, 242 parents reported having a child with an NDD diagnosis, whereas 205 parents who reported having children without an NDD diagnosis served as the comparison group. The Human Research Ethics Committee of The University of Hong Kong approved the study (#EA220008) and informed consent was obtained from all informants before data collection.

### Measures

#### Parental Stress

The Parental Stress Scale (PSS; Berry & Jones, 1995) was adopted to measure parental stress. In the validation study of the Chinese version PSS questionnaire (Cheung, 2000), one item was removed, resulting in a 17-item PSS questionnaire. The scale assesses parenting experience in both the positive aspect (e.g., “I find my child(ren) enjoyable.“; reverse scored) and negative aspect (e.g., “Having child(ren) leaves little time and flexibility in my life”). It adopts a 6-point Likert scale, with a higher score indicating higher stress. Internal consistency was excellent in the current study, *Cronbach’s α = 0.90.*

#### Parental Social Ecological Resilience

Social Ecological Resilience Scale (SERS; Wong et al., 2023) was used in the study. It contained 44 items that assess personal and interpersonal resilience in six subscales. The subscales include: (a) emotional flexibility (e.g., “After quarrelling with others, it is difficult for me to calm down.“; reverse scoring), (b) coping (e.g., “I can usually handle whatever comes my way.“), (c) self-kindness (e.g., “I’m kind to myself when I’m experiencing suffering.“), (d) common humanity (e.g., “I try to see my failings as part of the human condition.“), (e) family support (e.g., “We share responsibility in the family.“), and (f) social support (e.g., “My friends really try to help me.“). A higher score indicated higher resilience. Internal consistency was excellent in the current study, *Cronbach’s α = 0.91 (a*_*emotional flexibility*_*=0.90*,* a*_*coping*_*=0.92*,* a*_*self−kindness*_*=0.95*,* a*_*common humanity*_*=0.84*,* a*_*family support*_*=0.96*,* a*_*social support*_*=0.97).*

#### Children’s Internalising and Externalising Difficulties

Strengths and Difficulties Questionnaire: parent-report version for 2–4 year olds (SDQ; Goodman, 1997) was adopted. It comprises 20 items that measured children’s difficulties in four subscales. They are (a) emotional problems (e.g., “often unhappy, down-hearted or tearful.“), (b) peer problems (e.g., “picked on or bullied by other children.“), (c) conduct problems (e.g., “often has temper tantrums or hot tempers.“), and (d) hyperactivity (e.g., “restless, overactive, cannot stay still for long.“). Internalising difficulties was calculated by summing the scores of emotional problems and peer problems, and externalising difficulties was calculated as the summation of hyperactivity and conduct problems. Total difficulties were calculated by adding the scores of internalising and externalising difficulties. The Chinese version of the questionnaire was validated (Lai et al., 2010). Internal consistency was good in the current study, *Cronbach’s α = 0.81.*

### Statistical Analysis

All analyses were performed with IBM SPSS Statistics version 29. First, no missing data was found, and descriptive statistics were calculated. Second, to examine the predictive power of the six resilience factors on parental stress and children’s difficulties, two hierarchical multiple linear regression analyses were conducted, while controlling for parent age, parent gender, and child age. Third, by dividing the sample into the children-with-NDDs group (*n* = 242) and the children-without-NDDs group (*n* = 205), independent sample *t*-tests were conducted to compare parenting stress, resilience, and the difficulties of the children among the two groups. Fourth, the hierarchical multiple linear regression analyses described in the second step were replicated for each group separately: parents of children with NDDs and parents of children without NDDs. This allowed us to test whether the impact of resilience factors on parenting stress and children’s difficulties differed between the two groups, as hypothesized.

## Results

### Descriptive Statistics

Table 1 presents the demographic information of the participants. Of the 447 parents (mean age 38.8 ± 5.3), 88% were mothers and 93% were married. About half (55%) were full-time employees and 34% were homemakers. Most had either one child (50%) or two children (44%). More than half of them had children diagnosed with NDDs (54%), with ADHD (59%) and ASD (39%) at the most common diagnoses. Less than half of the parents (46%) reported that their children had no NDD diagnosis. The children’s mean (SD) age were 4.85 years old (2.18); 71% of them aged between 2 and 5 years.

**Table 1:** Demographic information of participants

		Total sample	Children with NDDs group	Children without NDDs group
		(*n* = 447)	(*n* = 242)	(*n* = 205)
Child age	Mean (SD)	4.85 (2.18)	5.18 (2.15)	4.46 (2.15)
Parent age	Mean (SD)	38.79 (5.33)	39.67 (5.80)	37.75 (4.51)
Parent gender				
	Male	52 (11.6%)	31 (12.8%)	21 (10.2%)
	Female	395 (88.4%)	211 (87.2%)	184 (89.8%)
Parent marital status				
	Single	7 (1.6%)	6 (2.5%)	1 (0.5%)
	Married	414 (92.6%)	218 (90.1%)	196 (95.6%)
	Divorced / separated	17 (3.8%)	11 (4.5%)	6 (2.9%)
	Widowed	5 (1.1%)	4 (1.7%)	1 (0.5%)
	Others	4 (0.9%)	3 (1.2%)	1 (0.5%)
Parent education level				
	Primary school or below	13 (2.9%)	13 (5.4%)	0
	Secondary education	92 (20.6%)	59 (24.4%)	33 (16.1%)
	Tertiary or above	342 (76.5%)	170 (70.1%)	172 (83.9%)
Parent employment status				
	Full-time employed	246 (55.0%)	119 (49.2%)	127 (62%)
	Part-time employed	39 (8.7%)	22 (9.1%)	17 (8.3%)
	Homemakers	153 (34.2%)	96 (39.7%)	57 (27.8%)
	Student	2 (0.4%)	1 (0.4%)	1 (0.5%)
	Unemployed / looking for jobs	7 (1.6%)	4 (1.7%)	3 (1.5%)
Monthly household income				
	USD1,282 and below	40 (8.9%)	25 (10.3%)	15 (7.3%)
	USD1,283–5,128	192 (43.0%)	118 (48.8%)	74 (36.1%)
	USD5,129–8,974	123 (27.5%)	57 (23.6%)	66 (32.2%)
	USD8,975 and above	92 (20.6%)	42 (17.4%)	50 (24.4%)
Household number of child(ren) aged below 18 years				
	One child	224 (50.1%)	116 (47.9%)	108 (52.7%)
	Two children	197 (44.1%)	109 (45.0%)	88 (42.9%)
	Three children	25 (5.6%)	16 (6.6%)	9 (4.4%)
	Four children or above	1 (0.2%)	1 (0.4%)	0
Type of NDDs				
	ADHD	143 (32.0%)	143 (59.1%)	
	ASD	95 (21.3%)	95 (39.3%)	
	Communication disorders	67 (15.0%)	67 (27.7%)	
	Specific learning disorder	64 (14.3%)	64 (26.4%)	
	Intellectual disability / global developmental delay	19 (4.3%)	19 (7.9%)	
The major caregiver of their child(ren)				
	Parents	216 (48.3%)	130 (53.7%)	86 (42.0%)
	Extended family members	113 (25.3%)	54 (22.3%)	59 (28.8%)
	Domestic helpers	107 (23.9%)	52 (21.5%)	55 (26.8%)
	Day-care centers / community centers	3 (0.7%)	2 (0.8%)	1 (0.5%)
	Others	8 (1.8%)	4 (1.7%)	4 (2.0%)

*Note* ADHD = attention deficit hyperactivity disorder; ASD = autism spectrum disorder; NDDs = Neurodevelopmental disorders

### Independent Sample t-tests

As illustrated in Table 2, parents of children with NDDs experienced significantly higher levels of parental stress, *t(445) = 7.34*, *p < .001*,* d = 0.70*. Similarly, children with NDDs exhibited more difficulties, *t(445) = 8.16*, *p < .001*,* d = 0.77*, including higher rates of internalising difficulties, *t(445) = 7.24*, *p < .001*,* d = 0.69*, and externalising difficulties, *t(445) = 6.22*, *p < .001*,* d = 0.63*. Furthermore, the parents of children with NDDs showed lower levels of emotion flexibility, *t(445) = -2.61*, *p < .01*,* d = − 0.25*, active coping, *t(445) = -3.23*, *p < .001*,* d = − 0.31*, self-kindness, *t(445) = -3.40*, *p < .001*,* d = − 0.32*, family support, *t(445) = 4.06*, *p < .001*,* d = − 0.39*, and social support, *t(445) = 3.44*, *p < .001*,* d = − 0.33*.

**Table 2:** Differences between families of children with and without NDDs

	Children with NDDs (*n* = 242)	Children without NDDs (*n* = 205)	Differences	
	Mean (SD)	Mean (SD)	*t*-value	Cohen’s *d*
Parents				
Parental stress	55.23 (11.69)	46.89 (12.31)	7.34***	0.70
Emotional flexibility	11.66 (3.97)	12.64 (4.00)	-2.61**	− 0.25
Coping	17.31 (4.47)	18.69 (4.55)	-3.23***	− 0.31
Self-kindness	8.71 (2.50)	9.48 (2.64)	-3.40***	− 0.32
Common humanity	9.74 (2.24)	10.06 (2.04)	-1.58	− 0.15
Family support	48.73 (8.79)	51.91 (7.60)	-4.06***	− 0.39
Social support	41.08 (11.03)	44.43 (9.23)	-3.44***	− 0.33
Children				
Total difficulties	17.91 (5.76)	13.61 (5.31)	8.16***	0.77
Internalizing difficulties	7.77 (3.33)	5.63 (2.85)	7.24***	0.69
Externalizing difficulties	10.14 (3.43)	7.98 (3.45)	6.22***	0.63

*Note* * *p* < .05, ** *p* < .01, *** *p* < .00. NDDs = Neurodevelopmental disorders

### Multiple Linear Regression Analyses

Table 3 presents the results of multiple linear regression analysis. The six resilience factors predicted 28% of variance in children’s symptoms and 36% in parental stress. Children’s symptoms were significantly predicted by emotional flexibility (*β = − 0.27*, *p < .001*), self-kindness (*β = − 0.22*, *p < .001*), family support (*β = − 0.17*, *p < .001*), and social support (*β = − 0.09*, *p < .05*). Parental stress was significantly predicted by emotional flexibility (*β = − 0.29*, *p < .001*), coping (*β = − 0.12*, *p < .01*), self-kindness (*β = − 0.15*, *p < .01*), family support (*β = − 0.18*, *p < .001*), and social support (*β = − 0.11*, *p < .01*).

**Table 3:** Multiple linear regression of parenting stress and difficulties of children on resilience

Dependent variables	Children’s difficulties	Parental stress
Beta (*β*)		
Emotional flexibility	− 0.27***	− 0.29***
Coping	− 0.01	− 0.12**
Self-kindness	− 0.22***	− 0.15**
Common humanity	0.06	− 0.04
Family support	− 0.17***	− 0.18***
Social support	− 0.09*	− 0.11*
Parent’s gender	− 0.12**	− 0.02
Parent’s age	0.08	0.02
Child’s age	0.00	0.01
R^2^	0.28	0.36
F-value	18.95***(9,437)	26.67***

*Note* * *p* < .05, ** *p* < .01, *** *p* < .001

### Subgroup Multiple Linear Regression Analyses

As shown in Table 4, several resilience factors significantly predicted parental stress and children’s difficulties among the two groups. Notably, emotional flexibility and family support demonstrated significant impacts on both children’s difficulties and parental stress among families of children with and without NDDs. Differential impacts were found among other resilience factors. Coping significantly predicted parental stress only for parents of children without NDDs (*β = − 0.24*, *p < .001*). Meanwhile, self-kindness predicted children’s difficulties (*β = − 0.29*, *p < .001*) and parental stress (*β = − 0.21*, *p < .01*) in the with NDD group.

**Table 4:** Multiple linear regression of families of children with and without NDDs

	Children with NDDs (*n* = 242)			Children without NDDs (*n* = 205)			
Dependent variables	Children’s difficulties	Parental stress		Children’s difficulties	Parental stress		
Beta (*β*)							
Emotional flexibility	− 0.31***	− 0.36***		− 0.22**	− 0.18**		
Coping	0.08	− 0.02		− 0.12	− 0.24***		
Self-kindness	− 0.29***	− 0.21**		− 0.09	− 0.08		
Common humanity	0.08	− 0.06		− 0.01	− 0.07		
Family support	− 0.15*	− 0.13*		− 0.18*	− 0.20**		
Social support	− 0.04	− 0.10		− 0.12	− 0.05		
Parent’s gender	− 0.13*	− 0.04		− 0.10	0.05		
Parent’s age	0.06	0.01		0.03	0.01		
Child’s age	0.05	− 0.04		− 0.15*	− 0.04		
R^2^	0.27	0.36		0.26	0.32		
F-value	9.36*** (9,232)	14.31***		7.55*** (9,195)	10.36***		

*Note* NDDs = Neurodevelopmental disorders.** *p* < .05, ** *p* < .01, *** *p* < .001

## Discussion

### Possible Cultural Factors Influencing Parenting Stress of Parents with Young Children with NDDs

Consistent with prior research (e.g., Fairthorne et al., 2015), our findings highlight that Chinese parents with young children with NDDs have a much higher elevation of parenting stress and more perceived symptoms in children’s internalising and externalising difficulties when compared to those parents with children without NDDs. A number of cultural factors may have accounted for the elevated parenting stress among our sample. First, Chinese culture emphasizes self-reliance and there is a tendency for Chinese people to rely on oneself or one’s very close family circle for support and help (Bond, 2010). Second, this issue is further intensified by the fact that there is a strong cultural stigma attached to having a child with special needs, which often are conceived as resulting from family misdeeds in the past or one’s own personal inadequacies (Ng & Ng, 2022). Consequently, these families may not seek help from others despite experiencing a huge amount of stress.

Given our samples are mainly younger parents (mean age 38.8 ± 5.3) with young children with NDDs (mean age 4.85 ± 2.18), earliest interventions aiming at educating and supporting this group of parents may be of paramount importance. For example, psychoeducation aimed at helping parents understand cultural influences and access available resources may not only alleviate feelings of shame and guilt but also empower them to seek support for themselves and their children. In addition, raising societal awareness of NDDs is essential for reducing stigma and fostering protective resilience factors within the community.

### Social Ecological Factors Influencing Parenting Stress and NDDs Children’s Difficulties

This study has adopted a social ecological perspective rather than the commonly adopted personal and psychological perspective in examining the resilience of parents with children with NDDs. As expected, our results revealed that parents with higher social ecological resilience have positive impacts on parenting stress and children’s behavioural difficulties. At the individual level, *emotional flexibility* and *self-kindness* were positively correlated with reduced parental stress and were negatively related to children’s symptoms. Apart from intrapersonal factors, we observed that at the interpersonal level, family and social support play significant roles in alleviating parenting stress and children’s symptoms. Together, these factors explained 28% of variance in children’s internalising and externalising difficulties and 36% in parenting stress. Indeed, the results underscore the importance of expanding the common personal and psychological perspective of resilience to including social and contextual factors in examining human resilience.

### Understanding the Varied Effects of Resilience Factors through a Context-Specific Perspective

However, what is noteworthy is, different types and levels of resilience appear to exert differential impacts on children’s internalising and externalising behaviours and parenting stress between parents with and without children with NDDs, except for *emotional flexibility* and *family support* which seems to be a universal resilience factor. From the literature, there is growing evidence suggesting that the flexibility with which was implement emotion regulation strategies might be key to our mental health (Kashdan & Rottenberg, 2010). In other words, any individual (parents in our specific study) who can effectively employ and switch to different emotion regulation strategies in handling different stressful situations would be able to maintain good mental health. What is interesting from this discussion is the idea that there are no absolute adaptive and maladaptive strategies, but effective situational and contextually motivated strategies that can facilitate constructive emotional regulations. Given this understanding, it is not surprising to see that our findings revealed emotional flexibility as a universal resilience factor. Indeed, it is suggested to include training in emotional flexibility in emotion regulation training programmes for parents with and without children with NDDs.

*Family support* appears to be another universal factor influencing parenting stress and children’s internalising and externalising behaviours. A substantial body of research has documented that mutual family support strengthens family functioning and buffers against the negative impacts of adversities (Bae & Kung, 2000; Walsh, 2003). Within the collectivist framework of Chinese society, family support may take on a greater importance for parents. Chinese parents often rely heavily on internal familial support systems, driven by cultural norms of family pride and a sense of shame in seeking external assistance (Bond, 2010). On the other hand, this also explains why *social support* did not exert any impact on parenting stress or children’s internalising and externalising behaviours. The reliance on family support and the shame and quilt surrounding having a child with NDD deter parents from seeking social support from outside of the family circle during adversities.

*Self-kindness* was only found to have a significant negative association with parent and child outcomes in families with NDDs, but not in families without NDDs. One way of understanding this result is, certain protective resilience factors such as self-kindness may be highly contextually motivated. For parents of children with NDDs, who experience high self-criticism and shame (Ng & Ng, 2022), self-kindness offers a way to view their situations without harsh self-judgment, promoting a compassionate and understanding attitude for themselves and their children during hardship (Reid et al., 2014). On the other hand, for parents who may not be experiencing such high stress, self-kindness as a mechanism may not hold the same importance. This result points towards the importance/need of further explore whether certain protective factors are contextually dependent, as this understanding would help guide policy makers and practitioners in developing more targeted strategies and programmes for the specific needs of the target group.

### Limitations

This study, while providing valuable insights into the resilience of Chinese families of children with NDDs, has certain limitations that should be acknowledged. Firstly, the research did not account for the type of NDDs, the length of time since diagnosis, or the potential impact of the child’s gender in influencing parenting stress and child’s emotions and behaviours. Considering that these factors can significantly influence parenting stress and child’s emotions and behaviours (Burnett et al., 2021; Miner & Clarke-Stewart, 2008; Sterba et al., 2007), our results should be interpreted with caution. Second, the cross-sectional design of this research limits the ability to infer causality between identified resilience factors and both parenting stress and the behavioral challenges observed in children. A longitudinal approach would be beneficial to understand these dynamics over time. Third, the identified resilience factors accounted for 28% of the variance in children’s internalising and externalising difficulties and 36% in parenting stress. This suggests that while these factors are significant, there may be other resilience factors that could provide additional explanatory power, such as the availability of community resources or other macro-level factors. Future research should consider incorporating a wider range of resilience factors, especially those at the community and societal levels.

Despite these limitations, social ecological resilience is a relatively novel concept in the resilience field, and this study represents the very few attempts to use a self-constructed and validated social ecological resilience scale (SERS) to understand different levels of resilience factors influencing the lives of parents of children with NDDs. Unlike previous resilience-related studies, our study has included both individual level factors (i.e. emotional flexibility, coping, self-kindness, and common humanity) and interpersonal level factors (i.e., family support and social support) to disentangle the multiple dimensions of resilience in influencing the parenting stress and children’s internalising and externalising behaviours. Our findings underscore the critical need to develop treatment plans that focus on enhancing a range of social ecological factors in families of children with NDDs. Additionally, the differential impacts of resilience factors between children-with- and without-NDDs groups indicated that certain protective factors may be contextually motivated. Policy makers and practitioners should consider developing context-specific strategies and programmes to help the focal target group.
